# Supplementary material for: Predicting the frequency of positive laboratory submissions for porcine reproductive and respiratory syndrome in Ontario, Canada, using autoregressive integrated moving average, exponential smoothing, random forest, and recurrent neural network
Source: PLoS One. 2025 Dec 31;20(12):e0339987. doi: 10.1371/journal.pone.0339987 (PMC12755821; doi:10.1371/journal.pone.0339987)
Supplement: S1 File — (DOCX) [file pone.0339987.s008.docx]

**S1 File. Extended description of time-series methods.**

In this section, the modeling approaches and assessment measures are described in greater detail. Particularly, this section provides the theoretical explanations of the following time-series regression models: 1) Autoregressive Integrated Moving Average (ARIMA); 2) Exponential Trend Smoothing (ETS); 3) Random Forest (RF); and 4) Recurrent Neural Network (RNN). A detailed review of the following accuracy measures used in forecasting includes: 1) Root Means Squared Error (RMSE); 2) Mean Absolute Error (MAE); 3) Mean Percentage Error (MPE); and 4) Mean Absolute Percentage Error (MAPE).

**Time-series regression models**

**Autoregressive integrated moving average (ARIMA)**

Let $\{Y_{t}\}$ be an arbitrary time series, i.e., a sequence of measurements of the same variable collected over time. Let $B$ be the lag operator that shifts $\{Y_{t}\}$ back $k$ periods, also known as a backshift operator, defined as $B^{k}Y_{t}=Y_{t-k}$. Then, in an AR process $\{Y_{t}\}$ of order *p* is defined as a linear aggregate of its *p* predecessors and a random term $Z_{t}$ and can be written as

$Y_{t}=\sum_{l=1}^{p} (\alpha_{l}Y_{t-l})+Z_{t}$,

where $\left\{ Z_{t} \right\}\sim WN(0,\sigma^{2})$; $WN$ is white noise with the mean 0 and the variance $\sigma^{2}$, and the $\alpha_{l}$ are weights. A moving average process of order *q* is a linear filter applied to current and *q* past random terms $\left\{ Z_{t} \right\}$and is defined as

$Y_{t}=Z_{t}+\sum_{j=1}^{q} (\beta_{j}Z_{t-j})$,

where $\left\{ Z_{t} \right\}\sim WN(0,\sigma^{2})$; $WN$ is white noise with the mean 0 and the variance $\sigma^{2}$, and the $\beta_{j}$ are weights. In terms of the lag operator $B$, an AR process of order $p$, AR$(p)$, and a MA process of order $q$, MA$(q)$, can be expressed as

$Z_{t}=(1-\sum_{l=1}^{p} \left( \alpha_{l}B^{t} \right){)Y}_{t}=\phi\left( B \right)Y_{t}$ ,

$Y_{t}=(1+\sum_{j=1}^{q} (\beta_{j}{B^{t}))Z}_{t}=\theta\left( B \right)Z_{t}$,

where $\phi\left( B \right)=1-\sum_{l=1}^{p} (\alpha_{l}B^{t})$ and $\theta\left( B \right)=1+\sum_{j=1}^{q} (\beta_{j}B^{t})$ are the polynomials of degree $p$ and $q$. The integrated part transforms $\{Y_{t}\}$ to account for nonstationary, i.e., to eliminate the effect of the trend on the value of a time series at different times. The transformation consists of taking the differences between lagged series observations. The first lag difference of $\{Y_{t}\}$ is defined by the expression $\left( 1-B \right)Y_{t}=Y_{t}{-Y}_{t-1}$. High order differences are defined by repeated application. That is, the second difference can be written as $\left( 1-B \right)^{2}Y_{t}=Y_{t}{-2Y_{t-1}+Y}_{t-2}$, and the $d$ difference can be defined by expression $\left( 1-B \right)^{d}Y_{t}$. Combining the three terms together, a time series $\{Y_{t}\}$ is an ARIMA process of order $p,d,q$: $\left\{ Y_{t} \right\}\sim ARIMA(p,d,q)$, with $\phi\left( 0 \right)=\theta\left( 0 \right)=1$. $ARIMA(p,d,q)$ is expressed as

$\phi\left( B \right)\left( 1-B \right)^{d}Y_{t}=\theta\left( B \right)Z_{t}$,

where after the $d^{th}$ difference of $\{Y_{t}\}$, the time series is a stationary ARMA process of order $p,q$; $\phi\left( B \right)=\sum_{l=1}^{p} (\alpha_{l}B^{t})$ and $\theta\left( B \right)=\sum_{j=1}^{q} (\beta_{j}B^{t})$; $B$ is a backshift operator. For more information, the reader is referred to [1,2].

The Akaike information Criterion (AIC) used for model selection is defined as

$AIC= -2\ln\left( L \right)+2k$,

where $L$ is the likelihood of the model, and $k$ is the number of estimated parameters. AIC balances model fit and complexity, penalizing models with more parameters. When comparing models, the one with the lower AIC is preferred, with differences greater than 10 considered strongly significant.

**Exponential smoothing (ETS)**

The exponential smoothing (ETS) methods have been used since the late 1950s [3–5]. These methods are based on smoothing random fluctuations and have desirable properties such as: (1) declining weights are placed on older data; (2) easy to compute; and (3) minimum data are required [4]. A predicted value of the average is obtained by computing a weighted average using the average of all past observations and the current value of the variable. The types of ETS models differ based on the type of time series, and they form an exponential forecasting system (EFS), or also known as the ETS state space models [6]. Every ETS model in the EFS is characterized by level (nonseasonal process), random effects (values caused by unpredictable events), noise (measurement error) and may or may not involve trend (a linear nonseasonal process) and seasonal (a periodic pattern) components. The detailed descriptions of the components can be found in [7]. The graphical representations of the effect of each component can be found in [5,7].

The ETS methods in the EFS taxonomy were proposed by Pegels [5] and extended by [6–13]. Here we refer to the same taxonomy as a framework for selecting amongst ETS methods using a three-letter code, which describes all possible trend and seasonal combinations. The first letter represents either an additive (A) or multiplicative (M) error. The second letter describes either none (N), additive (A), or damped (Ad) trend. The third letter represents either none (N), additive (A), or multiplicative (M) seasonal effect. Each ETS model in the EFS is labeled as ETS (Error, Trend, Seasonal).

Each model involves forecast and smoothing equations with their associated smoothing parameters. The first equation explains the observed data, while the second equation describes how unobserved errors, season, and trend components change over time.

Let $\{Y_{t}\}$ be an arbitrary time series, i.e., a sequence of measurements of the same variable collected over time. Define $x_{t}=(l_{t}, b_{t}, s_{t}, s_{t-1}, \ldots, s_{t-\left( m-1 \right)})$, where $l_{t}$ denotes the level, $b_{t}$ is the trend, $s_{t}$ describes the seasonal component, and $t$ denotes time. The ETS class of models can be written in general form as

Forecast equation: $Y_{t}=Hx_{t-1}+ \varepsilon_{t},$

Smoothing equation: $x_{t}=Fx_{t-1}+ G\varepsilon_{t},$

where $\left\{ \varepsilon_{t} \right\}\sim WN(0,\sigma^{2})$; $WN$ is white noise with the mean 0 and the variance $\sigma^{2}$; $H,F, G$ are coefficient matrices determined in [13]. For a simple exponential smoothing with additive errors, labeled as ETS (A, N, N), which is used for untrended and nonseasonal data, $H= F=1$ and $G=\alpha$ where $0<\alpha<2$ is the smoothing parameter for level of series [13]. The parameter space retains a stable model, and the stability region is identical to that for the equivalent invertible ARIMA model [13].

The equations for all ETS models in the EFS are given in [6,11]. The properties of ETS forecasting system are examined in [13]. The mathematical justifications of ETS models are referred to in [3,5,14].

The Bayesian Information Criterion (BIC) used for model selection process is defined as:

$BIC= -2\ln\left( L \right)+k ln(n)$,

where $L$ is the likelihood of the model, $k$ is the number of estimated parameters, and n is the number of observations. When comparing multiple models, a difference greater than 10 is considered strong evidence in favor of the model with lower BIC.

**Random forest (RF)**

Breiman’s RF algorithm [15] builds an ensemble of tree predictors such that each tree with branches and nodes is independently constructed from a different bootstrap sample, based on the principle of recursive partitioning where data space is recursively partitioned into smaller groups to minimize the amount of variation in each subspace. To minimize the chance of having trees with similar structures and thereby reducing the correlation among predictors while maintaining strength, Breiman applied two layers of randomness. He used the bootstrapping technique to construct trees and implemented random split selection where at each node the best split is selected from the $K$ randomly chosen predictors. The resulting forests are accurate regressors and are relatively robust to outliers and noise.

The RF regression begins by generating a training set for each tree by using bootstrap sampling from the original dataset. Each tree is trained on its respective bootstrap sample, while the remaining observations - referred to as the out-of-bag (OOB) samples - are used to evaluate model performance and estimate variable importance.

Trees are constructed by recursively splitting the data at each node. For each split, a random subset of $K$ predictors is selected from the full set of variables. Among the selected variables, all possible split values are evaluated to identify a predictor and threshold that yield the best reduction in the mean squared error (MSE). This process continues until a predefined stopping criterium is met (e.g., minimum node size and/or maximum tree depth). Each tree produces a prediction via a series of if-then statements, and the final RF prediction is obtained by averaging the prediction from all trees in the ensemble.

Model performance can be optimized by tuning the number of randomly selected predictors at each split and the total number of trees, using either cross-validation (CV) or OOB error as evaluation metrics. The CV approach involves partitioning the data into $K$ folds, training the model on $K-1$ folds, and validating it on the remaining fold. This process is repeated $K$ times (once for each fold), and the MSE is averaged across all validation sets to assess model performance.

Variables importance may also be assessed during this process by measuring the change in prediction error when each predictor is permuted or excluded. If a predictor contributes meaningfully to prediction accuracy, randomly permuting its values should lead to a substantial increase in the MSE. Conversely, if a predictor is irrelevant, permuting its values will have little to no effect on the performance. The percent increase in the MSE serves as a metric of variable importance [16].

The OOB error estimate is computed as the MSE between the observed values and the corresponding OOB predictions. For each observation, only the trees for which it was not included in the bootstrap sample are used to generate OOB prediction. The OOB MSE given by:

$MSE_{OOB}=\sum_{i=1}^{n} \frac{\left( Y_{i}- \hat{Y_{i}} \right)^{2}}{n}$*,*

where $Y_{i}$ is the observed value and $\hat{Y_{i}}$ is the average prediction for the $i^{th}$ observation, aggregated over only those trees in which it was out-of-bag.

Details on recursive partitioning and the RF algorithm are available in [15,17].

**Recurrent neural Networks (RNN)**

RNN are a class of artificial neural networks that are based on mathematical models of the brain. A neural network can be conceptualized as a network of neurons organized into layers (S6 Fig) [18–20]. RNNs integrate a fundamental component called a recurrent unit, which maintains a hidden state - an internal memory that enables the network to retain information from previous inputs. An output of RNN at any given time step depends on both the current input and the prior elements in the sequence (S6 Fig). The recurrent unit updates its hidden state based on the current input and the previous hidden state, allowing the network to learn temporal patterns and integrate past information into ongoing processing.

RNNs share parameters across time steps, using the same weights and biases at each point in the sequence. During training, gradient descent – an optimization algorithm used to find the minimum of a function – iteratively adjusts these weights and biases by estimating the direction of steepest descent to minimize the loss function.

RNNs operate as follows: at time step $t$, the input layer $X$ receives the current input in the sequence, which may be a scalar or a vector. Within the network architecture, a set of hidden units – often referred to as “Context units” or the “recurrent layer” – maintain recurrent state information. These units are distinct from the input and output layers and interact exclusively through internal recurrent connections (S6 -S7 Figs).

The context units apply an activation function (e.g., sigmoid or tanh) to produce outputs bounded within a specific range (typically 0 to 1 or -1 to 1). These outputs, along with the input, are passed to the hidden layer (or dense layer), which is equipped with its own activation function, weights, and biases (S7 Fig). In RNNs, the same set of parameters is shared across time steps, effectively unrolling a single hidden layer across time rather creating separate layers for each step.

At each time step, the hidden state is updated based on both the current input and the previous hidden state. This updated state is then used to generate the output and is also passed forward to serve as context for the next time step. This mechanism enables the network to retain memory of previous inputs and learn temporal dependencies.

During training, RNNs use the Backpropagation Through Time (BPTT) algorithm to compute gradients. BPTT unfolds the network along the time axis and computes the error gradients at each time step. The gradients are accumulated and used to update the shared weights via gradient descent, minimizing a predefined loss function.

For many time series applications, the Rectified Linear Unit (ReLU) is a commonly used activation function. It offers computational efficiency and helps mitigate the vanishing gradient problems [21]. ReLU is defined as the identity function for positive values and zero for negative values:

$$ReLU\left( x \right)=\max\left( 0, x \right)= \left\{ \begin{aligned} x, if x\geq0, \\ 0, otherwise. \end{aligned} \right.$$

**Long Short-Term Memory (LSTM)**

Long Short-Term Memory (LSTM) networks extend standard RNNs by introducing a memory cell and gating mechanisms that control the flow of information. This design helps preserve long-term dependencies and addresses the vanishing gradient problem. At each time step $t$, the LSTM computes the following:

$f_{t} =\sigma\left( W_{f}x_{t}+ U_{f}h_{t-1}+ b_{f} \right)$ forget gate,

$i_{t} =\sigma\left( W_{i} x_{t} + U_{i} h_{t-1} + b_{i} \right)$ input gate,

$o_{t} =\sigma\left( W_{o} x_{t} + U_{o} h_{t-1} + b_{o} \right)$ output gate,

$\tilde{c_{t}}=\tanh(W_{c} x_{t}+U_{c} h_{t-1}+b_{c})$ candidate cell state,

$c_{t} = f_{t}\odot c_{t-1} + i_{t}\odot\tilde{c_{t}}$ cell state update,

$h_{t} = o_{t}\odot\tanh(c_{t})$ hidden state/output,

where $x_{t}$ is the input vector, $h_{t-1}$ is the previous hidden state, $\sigma$ and $\tanh$ are the sigmoid and hyperbolic tangent activation functions, respectively. The operators $\odot$ and $+$ denote element-wise multiplication and addition. $W$, $U$, and $b$ represent learnable weight matrices and bias vectors. The gates modulate how much of the previous memory is retained, updated and exposed to the output.

**Gated Recurrent Unit (GRU)**

Gated Recurrent Units (GRU) simplify the LSTM architecture by combining the forget and input gates into a single update gate and omitting a separate cell state. GRUs are computationally efficient while maintaining performance in many sequential modeling tasks. At each time step $t$, the GRU computes:

$z_{t}=\sigma(W_{z} x_{t}+U_{z} h_{t-1} + b_{z})$ update gate,

$r_{t}=\sigma(W_{r} x_{t}+U_{r} h_{t-1} + b_{r}$) reset gate,

$\tilde{h_{t}}=\tanh((W_{h}x_{t}+U_{h} \left( r_{t}\odot h_{t-1}) +b_{h} \right)$ candidate hidden state,

$h_{t}= \left( 1-z_{t} \right) \odot h_{t-1} {+ z}_{t} \odot\tilde{h_{t}}$ new hidden state,

where $x_{t}$ is the input vector at time step $t$, $h_{t-1}$ is hidden state (output) from the previous time step. The update gate $z_{t}$ controls how much of the past information to retain, while the reset gate $r_{t}$ determines how to combine new input with past memory. Candidate hidden state $\tilde{h_{t}}$ is new memory content proposed by updating the hidden state, and $h_{t}$is the final hidden state at time step $t$, combining past and candidate memory. $\sigma$ is the sigmoid activation function that compresses values to (0,1), while $\tanh$ is the hyperbolic tangent activation function that squashes values into the range (-1,1). The operators $\odot$ and $+$ denote element-wise multiplication and addition. $W$, $U$, and $b$ represent learnable weight matrices and bias vectors.

Details on general deep learning sources, reader is referred [18–20,22,23].

**Assessment measures**

**Root Means Squared Error (RMSE)**

The RMSE is a scale-dependent measure and represents the sample standard deviation of the differences between predictions and observed values. The RMSE is defined as:

$$RMSE=\sqrt{\frac{1}{n}*\sum_{i}^{n} {{(P}_{i}-A_{i})}^{2}} ,$$

where $A_{i}, i=1, \ldots, n$ is the $i^{th}$ actual value; $P_{i}$ is the $i^{th}$ model predicted value by; $n$ is the test dataset size, the number of predicted values. The RMSE is commonly used because this measure is on the same scale as the data; however, the RMSE is sensitive to outliers. Therefore, we also considered the MAE, a robust scale-dependent measure.

**Mean Absolute Error (MAE)**

The MAE is considered as an unambiguous measure of average error magnitude [24] and is expressed as:

$$MAE=\frac{1}{n}*\sum_{i}^{n} {|P}_{i}-A_{i}|,$$

where $A_{i}, i=1, \ldots, n$ is the $i^{th}$ actual value; $P_{i}$ is the $i^{th}$ model predicted value by; $n$ is the test dataset size, number of predicted values. It has been reported that a forecasting modeling approach that minimizes the MAE will lead to forecasts of the median of time series, while minimizing the RMSE will lead to forecasts of the mean [25].

The MAE relative to the data range is defined as $\frac{MAE}{Data range}*100\%.$

To explore the accuracy of forecasts, we also studied scale-independent measures. These measures are the MPE and the MAPE. They are based on the percentage difference between the predictions and actual values. The MPE and the MAPE can be used only when the test data does not contain values of zero or close to zero. It also should be noted that percentage errors place a heavier penalty on positive errors for underestimated forecasts than on negative errors.

**Mean Percentage Error (MPE)**

The MPE is defined as:

$$MPE=\frac{1}{n}*\sum_{i}^{n} \left( \frac{P_{i}-A_{i}}{A_{i}}*100\% \right),$$

where $A_{i}, i=1, \ldots, n$ is the $i^{th}$ actual value; $P_{i}$ is the $i^{th}$ model predicted value by; $n$ is the test dataset size, number of forecasts.

Calculated MPE values can be positive or negative. If MPE >0, then the predicted values were higher than actual values, on average. Then, the forecasts were overestimated on average. If MPE < 0, then the predicted values were lower than the actual values on average, and the forecasts were underestimated, on average.

**Mean Absolute Percentage Error (MAPE)**

To disregard whether the forecasts were overestimated or underestimated, the MAPE measure, a relative measure, was computed. The MAPE is defined as:

$$MAPE=\frac{1}{n}{* \sum}_{i}^{n}\left| \frac{P_{i}-A_{i}}{A_{i}}*100\% \right|,$$

where $A_{i}, i=1, \ldots, n$ is the $i^{th}$ actual value; $P_{i}$ is the $i^{th}$ model predicted value by; $n$ is the test dataset size, number of predicted values.

A lower MAPE value indicates more accurate forecasts, while a higher MAPE value indicates less accurate forecasts. Detailed discussion about measures of forecast accuracy can be found in [24–26].

**References**

1. Brockwell P, Davis R. Time series: theory and methods. 2013.

2. Diggle P, Giorgi E. Time series; a biostatistical introduction. Oxford University Press; 2024.

3. Brown RG. Statistical forecasting for inventory control. New York: McGraw-Hill; 1959.

4. Holt CC. Forecasting seasonals and trends by exponentially weighted moving averages. Int J Forecast. 2004;20: 5–10. doi:10.1016/J.IJFORECAST.2003.09.015

5. Pegels CC. Exponential forecasting: some new variations. Manage Sci. 1969;15: 311–315. Available: https://www.jstor.org/stable/2628137

6. Hyndman RJ, Athanasopoulos G. Forecasting: principles and practice (3rd ed). 2021. Available: https://otexts.com/fpp3/

7. Stellwagen E, Goodrich R. Forecast pro. Business Forecast Systems; 2000. Available: https://www.pronosticoexperto.com/s/Forecast-Pro-Statistical-Reference-Manual.pdf

8. Ord JK, Koehler AB, Snyder RD. Estimation and prediction for a class of dynamic nonlinear statistical models. J Am Stat Assoc. 1997;92: 1621. doi:10.2307/2965433

9. Chatfield C, Yar M. Prediction intervals for multiplicative Holt-Winters. Int J Forecast. 1991;7: 31–37. doi:10.1016/0169-2070(91)90030-Y

10. Koehler AB, Snyder RD, Ord JK. Forecasting models and prediction intervals for the multiplicative Holt-Winters method. Int J Forecast. 2001;17: 269–286. Available: https://ideas.repec.org/a/eee/intfor/v17y2001i2p269-286.html

11. Hyndman RJ, Koehler AB, Snyder RD, Grose S. A state space framework for automatic forecasting using exponential smoothing methods. Int J Forecast. 2002;18: 439–454. doi:10.1016/S0169-2070(01)00110-8

12. Gardner ES. Exponential smoothing: the state of the art. J Forecast. 1985;4: 1–28. doi:10.1002/FOR.3980040103

13. Hyndman RJ, Akram M, Archibald BC, Hyndman RJ, Akram · M, Akram M, et al. The admissible parameter space for exponential smoothing models. SpringerRJ Hyndman, M Akram, BC ArchibaldAnnals of the Institute of Statistical Mathematics, 2008•Springer. 2008;60: 407–426. doi:10.1007/s10463-006-0109-x

14. Brown RG. Smoothing, forecasting and prediction of discrete time series. Englewood Cliffs, N.J.: Prentice-Hall, Inc.; 1963.

15. Breiman L. Random Forests. Mach Learn. 2001;45: 5–32. doi:10.1023/A:1010933404324

16. Svetnik V, Liaw A, Tong C, Wang T. Application of Breiman’s Random Forest to Modeling Structure-Activity Relationships of Pharmaceutical Molecules. International Workshop on Multiple Classifier Systems. Springer Berlin Heidelberg; 2004. doi:10.1007/978-3-540-25966-4_33

17. Kuhn M, Johnson K. Applied Predictive Modeling. New York: Springer; 2013.

18. Elman JL. Finding structure in time. Cogn Sci. 1990;14: 179–211. doi:10.1207/S15516709COG1402_1

19. Recurrent neural network - Wikipedia. Available: https://en.wikipedia.org/wiki/Recurrent_neural_network

20. Hopfield JJ. Neural networks and physical systems with emergent collective computational abilities. Proceedings of the National Academy of Sciences. 1982;79: 2554–2558. doi:10.1073/PNAS.79.8.2554

21. Dubey SR, Singh SK, Chaudhuri BB. Activation functions in deep learning: a comprehensive survey and benchmark. Neurocomputing. 2022;503: 92–108. doi:10.1016/J.NEUCOM.2022.06.111

22. Goodfellow I, Bengio Y, Courville A, Bengio Y. Deep learning. Cambridge: MIT press; 2016. doi:10.4258/HIR

23. Olah C. Understanding lstm networks. 2015 [cited 20 Jul 2025]. Available: https://research.google/pubs/understanding-lstm-networks/

24. Willmott CJ, Matsuura K. Advantages of the mean absolute error (MAE) over the root mean square error (RMSE) in assessing average model performance. Clim Res. 2005;30: 79–82. doi:10.3354/CR030079

25. Forecasting: Principles and Practice (2nd ed). [cited 29 Nov 2023]. Available: https://otexts.com/fpp2/

26. Hyndman RJ, Koehler AB. Another look at measures of forecast accuracy. Int J Forecast. 2006;22: 679–688. doi:10.1016/J.IJFORECAST.2006.03.001
