# Supplementary material for: Predicting the frequency of positive laboratory submissions for porcine reproductive and respiratory syndrome in Ontario, Canada, using autoregressive integrated moving average, exponential smoothing, random forest, and recurrent neural network
Source: PLoS One. 2025 Dec 31;20(12):e0339987. doi: 10.1371/journal.pone.0339987 (PMC12755821; doi:10.1371/journal.pone.0339987)
Supplement: Attachment — Submitted filename: Response to Reviewers.pdf [file pone.0339987.s010.pdf]

UNIVERSITY of GUELPH

November 6, 2025

Ahmad Salimi
Academic Editor
PLOS ONE

RE: PONE-D-25-39776

Dear Shaman,

We are submitting the first revised version of our original manuscript: "Predicting the frequency of positive laboratory submissions for porcine reproductive and respiratory syndrome in Ontario, Canada, using autoregressive integrated moving average, exponential smoothing, random forest, and recurrent neural network" for possible publication in PLOS ONE. We followed your advice and have addressed all the concerns raised during this review process. We thank you and the reviewers for the comments and suggestions. Below, we address journal requirements and each point raised by the academic reviewers:

Editor's comments:

1. Please ensure that your manuscript meets PLOS ONE's style requirements, including those for file naming.
2.

Author response:
We have reviewed the PLOS ONE's style requirements and ensured our manuscript fully complies with them, including file naming and formatting guidelines.

2. Please note that PLOS One has specific guidelines on code sharing for submissions in which author-generated code underpins the findings in the manuscript. In these cases, we expect all author-generated code to be made available without restrictions upon publication of the work. Please review our guidelines at https://journals.plos.org/plosone/s/materials-and-software-sharing#loc-sharing-code and ensure that your code is shared in a way that follows best practice and facilitates reproducibility and reuse.

Author response:

We have reviewed the PLOS ONE guidelines on code sharing and will ensure that our author-generated code is shared, if required, in accordance with these recommendations to promote reproducibility and reuse.

3. Thank you for stating the following financial disclosure. Please state what role the funders took in the study. If the funders had no role, please state: "The funders had no role in study design, data collection and analysis, decision to publish, or preparation of the manuscript."

If this statement is not correct you must amend it as needed.

Please include this amended Role of Funder statement in your cover letter; we will change the online submission form on your behalf.

Author response:
Please include the following Role of Funder statement:
"The funders had no role in study design, data collection and analysis, decision to publish, or preparation of the manuscript."

4. We note that you have indicated that there are restrictions to data sharing for this study. PLOS only allows data to be available upon request if there are legal or ethical restrictions on sharing data publicly. For more information on unacceptable data access restrictions, please see http://journals.plos.org/plosone/s/data-availability#loc-unacceptable-data-access-restrictions.

Before we proceed with your manuscript, please address the following prompts:

a) If there are ethical or legal restrictions on sharing a de-identified data set, please explain them in detail (e.g., data contain potentially identifying or sensitive patient information, data are owned by a third-party organization, etc.) and who has imposed them (e.g., a Research Ethics Committee or Institutional Review Board, etc.). Please also provide contact information for a data access committee, ethics committee, or other institutional body to which data requests may be sent.

Author response:

Data used for this study cannot be shared publicly due to our REB application. Part of the REB application is also founded on the confidentiality agreement between the principal investigator and the laboratory providing access to the data. They are confidential and, in some cases, could be identifiable even after de-identification. Contact information for our institutional REB committee is reb@uoguelph.ca.b) If there are no restrictions, please upload the minimal anonymized data set necessary

to replicate your study findings to a stable, public repository and provide us with the relevant URLs, DOIs, or accession numbers. For a list of recommended repositories, please see

https://journals.plos.org/plosone/s/recommended-repositories. You also have the option of uploading the data as Supporting Information files, but we would recommend depositing data directly to a data repository if possible.

We will update your Data Availability statement on your behalf to reflect the information you provide.

Author response:
Please retain the current Data Availability statement. The data cannot be shared publicly due to confidentiality.

5. Thank you for stating the following in the Acknowledgments Section of your manuscript:

"This project was funded by the University of Guelph's Food From Thought program. We would like to thank the members of the Ontario Animal Health Network swine group for their feedback and advice."

We note that you have provided additional information within the Acknowledgements Section that is not currently declared in your Funding Statement. Please note that funding information should not appear in the Acknowledgments section or other areas of your manuscript. We will only publish funding information present in the Funding Statement section of the online submission form.

Please remove any funding-related text from the manuscript and let us know how you would like to update your Funding Statement. Currently, your Funding Statement reads as follows:

"The author who received funding: ZP

Grant number: 499163

This project was funded by the University of Guelph's Food From Thought program

The author who received funding: MS

Grant number: 499170

This project was funded by the University of Guelph's Food From Thought program”

Please include your amended statements within your cover letter; we will change the online submission form on your behalf.

Author response:
All funding-related text has been removed from the Acknowledgements section and the main manuscript. We would like to change the current Funding Statement as the following:

This project was supported by the Food from Thought program at the University of Guelph, funded through the Canada First Research Excellence Fund (Grant number: 499163, the author who received funding: ZP; Grant number: 499170, The author who received funding: MS), and by the Ontario Agri-Food Innovation Alliance (grant number: 100022, the author who received funding: ZP).

6. If the reviewer comments include a recommendation to cite specific previously published works, please review and evaluate these publications to determine whether they are relevant and should be cited. There is no requirement to cite these works unless the editor has indicated otherwise.

Author response:
No such recommendations were made by the reviewers.

Reviewer #1

1. Data and Scope

Reviewer comment:
Could you clarify why both diagnostic and monitoring submissions were included? Would separating them improve model accuracy?

Author response:
Thank you for this valuable comment. We fully agree with the rationale for separating diagnostic and monitoring submissions to improve model accuracy. This approach was indeed part of initial consideration before conducting a more detailed data analysis. In our previous publication (Petukhova T. et al., *Front Vet Sci*. 2025; 12:1528422. doi:10.3389/FVETS.2025.1528422), we specifically investigated the impact of splitting diagnostic and monitoring data. The relevant findings from that study are quoted as follows “In addition, a pattern in the number of monitoring submissions submitted per month was observed, where three distinct

periods were visible (Figure 1) with abrupt changes in the number of submissions that coincided with the time that the calendar year ends and starts. The period until 2017 was characterized by a progressively higher number of submissions over time (mean = 38.7, sd = 15.9), followed by a generally lower number of submissions between January 2017 and December 2021 (mean = 27.3 sd = 9.7), and a subsequent increase in the number of monitoring submissions starting in January 2022 (mean = 65, sd = 14.7) (Figure 1). Similar patterns were noted in monitoring submissions in both nursery (Supplementary Figure 1) and grower/finisher pigs (Supplementary Figure 2). An interesting pattern was also observed among submissions for suckling pigs: while the number of all submissions increased over time and particularly since the start of 2020 (Supplementary Figure 3), the number of monitoring submissions in this production class was low until 2022 (Supplementary Figure 4)".

In the discussion of previously published paper, we specifically made a note about this observation by stating "Research into understanding how end users define a monitoring vs. a diagnostic submission, and whether they adhere to their definition when submitting samples for pathogen testing, is warranted before relying too much on its interpretation. Classification into monitoring and diagnostic submissions as the two basic categories is sensible, but it would be prudent to understand practices that contribute to defining these submission types, and motivations of people who submit samples to indicate certain submission type...."

In brief, we believed that by using all the data, we would be better at investigating any short- or long-term trends, as there were clearly factors that impacted such classification that we could not capture.

Nonetheless to address your comment fully, we applied time series modeling separately to datasets containing (i) only diagnostic submissions, and (ii) both diagnostic and monitoring submissions to assess whether model accuracy differed substantially between them. The result indicated that using only diagnostic submissions led to slight improvements in model accuracy based on RMSE and MAE metrics (but not based on the MPE and MAPE metrics, which were higher). However, these differences were marginal and inconsistent across evaluation approaches. For example, in the first evaluation approach (RF model), diagnostic-only data yielded RMSE = 4.3, MAE = 3.3, MPE = 7.9%, MAPE = 40.3%, whereas modeling both diagnostic and monitoring submissions resulted in RMSE = 5.0, MAE = 3.9, MPE = -4.4%, MAPE = 36.5%. In the second evaluation approach, the corresponding values were RMSE = 2.92, MAE = 2.91, MPE = 4.05%, MAPE = 36.5% (diagnostic-only) and RMSE = 3.79, MAE = 3.11, MPE = 0.36%, MAPE = 34.29% (combined data).

Thus, given that excluding monitoring submissions did not provide substantial gains with all the issues that we identified in the previous paper, and with potentially introducing bias and uncertainty due to inconsistent reporting of submission reasons, we opted to include both diagnostic and monitoring data in our final modeling. This decision is consistent with previous work demonstrating the value of integrating both submission types for comprehensive surveillance (Petukhova T. et al., *Front Vet Sci*. 2025; 12:1528422. doi:10.3389/FVETS.2025.1528422). We have clarified this point in the Discussion section of the revised manuscript (lines 582-596 and 650-658).

Reviewer comment:
Were farm-specific factors (e.g., size, location, climate) considered in the analysis?

Author response:
Farm-specific factors (e.g., size, location, climate) were not included in the analyses because this submission information was not available in the dataset. We acknowledge that these variables could potentially influence disease dynamics, and we plan to incorporate them in future work if such data become accessible. We add this information as a limitation in the manuscript (see lines 668-671 of the revised manuscript).

2. Models

Reviewer comment:
How were hyperparameters optimized for the RF and RNN models? Was a systematic tuning procedure applied?

Author response:
The optimal hyperparameters for the RF were identified using the tuneRF() function and subsequently applied in the RF regression procedure. For the RNN model, hyperparameter tuning was performed through a systematic search process within the *keras* framework. Model performance was evaluated using MAE and MSE metrics, and early stopping was applied to prevent overfitting. These clarifications have been added to the revised manuscript (lines 226-230 and 300-309).

Reviewer comment:
Did you explore transformations (e.g., log or power) for the count data to improve ARIMA model performance?

Author response:

In fitting the ARIMA model, we used the auto.arima() function with the Box-Cox transformation parameter set to "auto". This allowed the function to automatically select an appropriate variance-stabilizing transformation based on the data, helping to improve ARIMA model performance. We have clarified this point in the Methods section (see lines 160-164 of the revised manuscript). We also reported which transformation was selected by the function in the *Results* section (line 450).

3. Interpretation of Results

Reviewer comment:
Given the relatively high MAPE (~35–42%), what are the practical implications for PRRSV surveillance?

Author response:
We acknowledge that the MAPE values of ~35-42% indicate moderate predictive error in our models. Nevertheless, all four models effectively captured key temporal trend, and some provided early indications of increasing activities, supporting their potential utility for predictive purposes in surveillance. However, the current level of accuracy remains insufficient for implementation in regular interactive animal pathogen dashboards (IAPD). Further refinement of the model and/or processing of input data is needed to improve performance and provide clearer indications of incident cases submitted for diagnostic purposes. The precision and standardization of submission records, aggregating data at a larger temporal scale (e.g., monthly) or incorporating additional farm-level variables may further improve predictive performance in future studies. We added this practical implication in the Discussion section of the revised manuscript (lines 660-676).

Reviewer comment:
Why was no clear seasonal pattern observed, while previous studies indicate higher transmission in colder months?

Author response:
In our study, the modeled data consisted of test results from samples submitted throughout the years, showing substantial variability across all seasons. Descriptive statistics and graphical assessments indicated large week-to-week fluctuations in the number of positive results during winter (e.g., 2-17 positives in week 1, 3-17 in week 48, 3- 23 in week 49), spring (e.g., 2-21 in week 11, 0-20 in week 13, 4-23 in week 17), summer (e.g., 1-18 in week 25, 0-17 in week 29, 3-16 in week 33), and fall (e.g., 2-17 in week 44). The variability in the positive counts also increased notably since 2017.

This high variability across seasons likely obscured any clear seasonal signal in the data. Several potential factors may have contributed to the variability, including frequent testing conducted across different phases of disease investigation, management, intervention, or monitoring, both during and after herd closure. It is also possible that seasonal transmission patterns vary by geographic region and are influenced by local climate conditions, changes in farm management, or biosecurity measures over time. As shown in Figure 2, the seasonal component explained only 9% of the variance, suggesting that seasonality was weak relative to other sources of variation, which likely explains why the ARIMA and ETS models did not detect a pronounced seasonal pattern. In the revised manuscript, we provided more details in the Result section and expanded the discussion to clarify possible reasons why seasonal patterns were not detected by the time-series models (lines 376-389, 572-580, and 597-601).

4. Limitations and Applicability

Reviewer comment:
Do the authors believe the results are generalizable to other provinces or countries with different conditions?

Author response:
We acknowledge that the results are primarily reflect the epidemiological and management conditions in Ontario, Canada, where the data were collected. Therefore, direct generalization to other provinces or countries should be done with caution, as differences in swine production systems, biosecurity practices, and surveillance intensity may influence PRRSV dynamics. Nevertheless, the analytical framework and modeling approaches presented in this study can be readily applied to similar datasets from other regions to assess local patterns and inform surveillance strategies under different conditions. We have clarified this point in the Discussion section of the revised manuscript (lines 677-682).

Reviewer comment:
Have ensemble or hybrid modeling approaches been considered to potentially enhance predictive accuracy?

Author response:
We appreciate the reviewer's suggestion regarding ensemble or hybrid modeling approaches. In this study, we focused on comparing two classical, one machine learning, and one deep learning time series models to characterized PRRSV frequency dynamics. We selected ARIMA and ETS models to represent classical

time series methods because they are widely used, transparent, interpretable, and computationally efficient. RF and RNN models were included as flexible approaches capable of capturing complex, nonlinear patterns. We agree that exploring ensemble or hybrid approaches, including advanced machine learning or deep learning architectures, could potentially enhance predictive accuracy. However, our analyses indicated that the PRRSV submission data contained substantial irregularly spaced outliers across all seasons, which masked temporal signals. Even after variance-stabilizing transformation, predictions remained challenging. Consequently, while ensemble and hybrid models could be applied, their benefits are uncertain in the presence of such irregular dynamics. Nevertheless, we plan to consider these methods in future work (see lines 668-674 of the revised manuscript).

Potential Weaknesses / Issues

1. Methodology

Reviewer comment:
Limited explanation of submission type selection and its potential impact on model outcomes.

Author response:
We have expanded the explanation of submission type selection and clarified its potential impact on model outcomes in point in the *Discussion* section of the revised manuscript (lines 582-596 and 650-658).

Reviewer comment:
Insufficient details on hyperparameter optimization for RF and RNN models.

Author response:
We have added additional details on the hyperparameter optimization approaches used for both the RF and RNN models in the *Materials and Methods* section of the revised manuscript (lines 226-230 and 300-309).

Reviewer comment:
No discussion of handling zero values in count data for ARIMA modeling.

Author response:
We quantified the proportion of zero values, characterized and discussed them in the *Results* and *Discussion* sections of the revised manuscript (lines 374-376, 568-572). Descriptive analyses indicated that the few zero counts observed in some weeks (1.2% of the data) likely reflected variability in the submission process

rather than a true absence of disease. Given their small proportion, these zero values did not affect the time series models (e.g., after differencing with ARIMA, the distribution of residuals appeared reasonably symmetric).

2. Prediction Accuracy

Reviewer comment:
High MAPE values (35–42%) indicate moderate predictive performance; this should be discussed in terms of practical surveillance applications.

Author response:
We have addressed this comment by discussing the moderate forecasting performance (MAPE ≈ 35-42%) and its practical implications for surveillance applications in the revised manuscript (lines 660-676).

Reviewer comment:
ARIMA and ETS models tend to overpredict; reasons for these biases are not fully explained.

Author response:
We appreciate the reviewer's observation. In the revised manuscript, we expanded and clarified the discussion of the possible reasons for the overprediction tendencies in the ARIMA and ETS models (lines 615-626).

3. Results Interpretation

Reviewer comment:
Lack of clear seasonal patterns contradicts prior epidemiological knowledge; explanations are speculative and not deeply analyzed.

Author response:
We acknowledge the reviewer's point regarding the apparent absence of clear seasonal pattern. In the revised manuscript, we provided more details in the Result section and expanded the discussion to clarify possible reasons why seasonal patterns were not detected by the time series models, including frequent testing conducted across different phases, data limitations, model sensitivity, regional variability (Fig 2, lines 376-389, 572-580, and 597-601).

Reviewer comment:
Some discussion sections are lengthy and could be clarified for better readability.

Author response:

In response to reviewer's comment, we have carefully revisited the Discussion to clarify key points and improve readability, shortening some sections for conciseness while maintaining scientific content.

4. Generalizability

Reviewer comment:
Data are limited to one province; environmental and management factors are not fully incorporated, limiting broader applicability.

Author response:
We recognize that the study is based on data from Ontario, Canada, and that environmental and management factors could not be incorporated because they were not available. While this limits broader applicability, the analytical framework and analytical approaches we used can be applied to similar datasets from other regions or countries to evaluate local patterns and inform surveillance strategies. This clarification has been added to the *Discussion* section of the revised manuscript (lines 668-682).

Reviewer #2

1. **Reviewer comment:**

The rationale for selecting only ARIMA, ETS, RF, and RNN is unclear. The authors should clarify why more advanced or hybrid methods (e.g., Prophet, XGBoost, or ensemble models) were not considered.

Author response:
We thank the reviewer for this comment. We selected ARIMA and ETS models to represent classical time series methods because they are widely used, transparent, interpretable, and computationally efficient. RF and RNN models were included as flexible approaches capable of capturing complex, nonlinear patterns, as noted in the Discussion section (revised manuscript lines 560-563).

Our analyses indicated that the PRRSV submission data contained substantial irregularly spaced outliers across all seasons, which masked temporal signals. Even after variance-stabilizing transformation, predictions remained challenging. Consequently, while advanced and hybrid models (e.g., Prophet, XGBoost, or ensemble models) could be applied, their benefits are uncertain in the presence of such irregular dynamics. We, therefore, focused on the selected models and suggest that exploring more advanced methods would be a useful direction for future research (revised manuscript lines 650-652 and 668-674).

2. **Reviewer comment:**

The reported forecasting error (MAPE ≈ 35%) is relatively high. Conclusions regarding the suitability of RF as the "best model" should be tempered to reflect this limitation.

Author response:

We acknowledge that although RF model was the most accurate among the time-series methods evaluated (ARIMA, ETS, and RNN), it still exhibited relatively high prediction errors (MAPE ≈ 35%). Accordingly, we have revised the *Discussion* section to temper our conclusions, stating that the RF model demonstrated the highest predictive accuracy. However, its current level of accuracy remains insufficient for implementation in regular interactive animal pathogen dashboards (IAPD). Further refinement of the model and/or processing of input data is needed to improve performance and provide clearer indications of incident cases submitted for diagnostic purposes

Therefore, before considering such a forecasting tool, future work should focus on enhancing forecasting efficacy by improving the precision and standardization of submission records, including combinations of multiple methods, exploring temporal aggregations (e.g., monthly data), or incorporating farm-level variables. Such efforts could help determine whether the observed performance levels are sufficient for operational use within the regular PRRSV surveillance system (revised manuscript lines 663-676).

3. **Reviewer comment:**

The study did not detect seasonal patterns, which contradicts previous reports. Stronger statistical tests or clearer scientific explanations are required to justify this discrepancy.

Author response:

Thank you for raising this important. Although previous studies have reported seasonality, our analysis of the submitted test results revealed substantial week-to-week variation in positive counts throughout the years across all seasons (e.g., 2-17 positives in week 1, 3-17 in week 48, 3- 23 in week 49), spring (e.g., 2-21 in week 11, 0-20 in week 13, 4-23 in week 17), summer (e.g., 1-18 in week 25, 0-17 in week 29, 3-16 in week 33), and fall (e.g., 2-17 in week 44). Notably, this variability has grown since 2017. This high variability across seasons likely obscured any clear seasonal signal in the data. Several potential factors may have contributed to the variability, including frequent testing conducted across different phases of disease investigation, management, intervention, or monitoring during and after herd closure), changes in climate conditions, farm management practices, and biosecurity measures over time. As shown in Figure 2, the seasonal component

explained only 9% of the variance, suggesting that seasonality was weak relative to other sources of variation, which likely explains why the ARIMA and ETS models did not detect a pronounced seasonal pattern.

We have updated the *Result* section and expanded the *Discussion* to clarify these points and to suggest plausible explanations why seasonal patterns were not detected by the time series models (see revised manuscript, lines 376-389, 572-580, and 597-601).

4. **Reviewer comment:**

The combination of diagnostic and monitoring submissions may have introduced noise. A sensitivity analysis restricted to diagnostic data would strengthen the study.

Author response:
We appreciate the reviewer's insightful comment. We agree that the combination of diagnostic and monitoring submissions may have introduced noise. Separating diagnostic and monitoring submissions to improve model accuracy was indeed part of initial consideration before conducting a more detailed data analysis. In our previous publication (Petukhova T. et al., *Front Vet Sci*. 2025; 12:1528422. doi:10.3389/FVETS.2025.1528422), we specifically investigated the impact of splitting diagnostic and monitoring data. The relevant findings from that study are quoted as follows "In addition, a pattern in the number of monitoring submissions submitted per month was observed, where three distinct periods were visible (Figure 1) with abrupt changes in the number of submissions that coincided with the time that the calendar year ends and starts. The period until 2017 was characterized by a progressively higher number of submissions over time (mean = 38.7, sd = 15.9), followed by a generally lower number of submissions between January 2017 and December 2021 (mean = 27.3 sd = 9.7), and a subsequent increase in the number of monitoring submissions starting in January 2022 (mean = 65, sd = 14.7) (Figure 1). Similar patterns were noted in monitoring submissions in both nursery (Supplementary Figure 1) and grower/finisher pigs (Supplementary Figure 2). An interesting pattern was also observed among submissions for suckling pigs: while the number of all submissions increased over time and particularly since the start of 2020 (Supplementary Figure 3), the number of monitoring submissions in this production class was low until 2022 (Supplementary Figure 4)".

In the discussion, we specifically made a note about this observation by stating "Research into understanding how end users define a monitoring vs. a diagnostic

submission, and whether they adhere to their definition when submitting samples for pathogen testing, is warranted before relying too much on its interpretation. Classification into monitoring and diagnostic submissions as the two basic categories is sensible, but it would be prudent to understand practices that contribute to defining these submission types, and motivations of people who submit samples to indicate certain submission type...."

In brief, we believed that by using all the data, we would be better at investigating any short- or long-term trends, as there were clearly factors that impacted such classification that we could not capture.

Nonetheless to address your comments fully, we applied time series modeling separately to datasets containing (i) only diagnostic submissions, and (ii) both diagnostic and monitoring submissions to assess whether model accuracy differed significantly between them. The results indicated that using only diagnostic submissions led to a slight improvement in model accuracy based on RMSE and MAE metrics, but the differences were marginal and inconsistent across evaluation approaches. For example, in the first evaluation approach (RF model), diagnostic-only data yielded RMSE = 4.3, MAE = 3.3, MPE = 7.9%, MAPE = 40.3%, whereas modeling both diagnostic and monitoring submissions resulted in RMSE = 5.0, MAE = 3.9, MPE = -4.4%, MAPE = 36.5%. In the second evaluation approach, the corresponding values were RMSE = 2.92, MAE = 2.91, MPE = 4.05%, MAPE = 36.5% (diagnostic-only) and RMSE = 3.79, MAE = 3.11, MPE = 0.36%, MAPE = 34.29% (combined data).

Thus, given that excluding monitoring submissions did not provide substantial gains with all the issues that we identified in the paper, and with potentially introducing bias and uncertainty due to inconsistent reporting of submission reasons, we opted to include both diagnostic and monitoring data in our final modeling. This decision is consistent with previous work demonstrating the value of integrating both submission types for comprehensive surveillance (Petukhova T. et al., *Front Vet Sci*. 2025; 12:1528422. doi:10.3389/FVETS.2025.1528422). We have clarified this point in the Discussion section of the revised manuscript (lines 582-596 and 650-658).

5. **Reviewer comment:**

Given the relatively small dataset, the use of RNN may not be appropriate. This limitation should be more clearly discussed.

Author response:

We acknowledge that our dataset - comprising 654 weekly observations - is relatively small for training deep learning models such as RNNs. Consequently, we

took particular care to mitigate overfitting. Our rationale for including an RNN model was to assess whether it could capture nonlinear and complex temporal dependencies that may not be adequately modeled by classical time-series models such as ARIMA and ETS as well as simpler machine learning methods like RF.

To ensure that RNN generalized rather than memorized the data, we implemented several regularization techniques, including dropout, recurrent dropout, and early stopping along with windowing (e.g., training on overlapping 12-week input windows to effectively increase the number of training samples). Despite the relatively small dataset, the RNN model demonstrated better predictive performance than the fitted ARIMA and RF. We have now explicitly discussed the RNN limitation and the implications for generalizability in the revised manuscript (lines 627-641).

We hope that the editions made to the manuscript to address all of the concerns expressed by the reviewers are considered satisfactory. We believe that the contents and readability of the manuscript have been greatly improved in this revised version. We thank you for your consideration.

Sincerely,

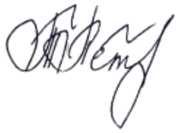

Tatiana Petukhova
Department of Population Medicine
Ontario Veterinary College
University of Guelph
Guelph ON N1G 2W1
Canada

E-mail: tpetukho@uoguelph.ca
